# An Examination of Who Is Eligible and Who Is Receiving Bariatric Surgery in England: Secondary Analysis of the Health Survey for England Dataset

**DOI:** 10.1007/s11695-019-03977-3

**Published:** 2019-05-25

**Authors:** Daniel Desogus, Vinod Menon, Rishi Singhal, Oyinlola Oyebode

**Affiliations:** 1 Warwick Medical School, Coventry, UK; 2 University Hospitals of Coventry and Warwickshire NHS Trust, Coventry, UK; 3 Heart of England NHS Trust, Birmingham, UK

**Keywords:** Bariatric surgery, Clinical guidelines, Population prevalence, Treatment demand, Penetration

## Abstract

**Background:**

Over 2 million people in England were estimated to be eligible for bariatric surgery in 2006. In 2014, clinical guidelines were updated, widening potential eligibility, meanwhile, obesity prevalence rose. However, numbers receiving surgery decreased, and concerns exist of inequalities in access between population groups. This study is aimed at estimating the number of adults eligible for surgery in England and to compare demographics with those that receive surgery.

**Methods:**

BMI and comorbidity status were used to determine eligibility for bariatric surgery within participants of the 2014 Health Survey for England dataset (6938 adults), based on the National Institute of Health and Care Excellence guidelines. Results were scaled up using national population estimates. The demographics of eligible participants were compared against 2014/2015 hospital episode statistics for sex and age group using a chi-squared analysis.

**Results:**

Of the total population of England, 7.78% (95% CI 7.1–8.6%), or 3,623,505 people, could have been eligible for bariatric surgery in 2014; nearly a million more than if previous guidelines applied. Eligibility peaked at ages 45–54, with most in the 35–64 age group (58.9%). 58.4% of those eligible were women. Patients receiving surgery were far more likely to be female than male (76.1%) and the distribution skewed towards younger ages when compared with those eligible.

**Conclusion:**

Bariatric surgery may benefit many people in England; significant investment is required so that service provision is adequate for demand. Differences between demographics of those eligible and receiving surgery may be explainable; however, the potential health inequality should be investigated.

## Introduction

Obesity is a worldwide health problem, being linked to reduced life expectancy and multiple comorbidities [1, 2]. An analysis of the 2016 Health Survey for England (HSE) data found that 26% of men and 27% of women were obese in the UK (defined as BMI of 30 kg/m^2^ or above) [3]. Recent estimates for the cost to the NHS for overweight and obese patients estimate it to be around £6.05 billion per year [4]. Effective prevention and treatment is needed to reduce these numbers, to help improve the life expectancy and quality of life of patients, and to reduce the economic burden on the NHS.

In 2014, the UK National Institute for Clinical Excellence (NICE) guidelines for “Obesity: identification, assessment and management” were updated from the 2006 guidelines. The current NICE guidelines recommend bariatric surgery as a treatment for those with a body mass index (BMI) above 40 kg/m^2^, or between 35 and 39.5 kg/m^2^ if other significant diseases are present that could be improved with weight loss. The recommendation for surgery in these groups is dependent on other factors, such as trying all appropriate non-surgical measures (usually behavioural interventions and potentially pharmacological interventions). Patients with a BMI above 50 kg/m^2^ are eligible for bariatric surgery regardless of whether they have tried lifestyle or drug interventions. These sections of the guidelines are the same as in the previous guidelines. The new guidance introduced allows bariatric surgery as a first-line treatment for those with recent-onset type two diabetes and a BMI of 30–34.9 kg/m^2^, with an expedited assessment recommended if they have a BMI above 35 kg/m^2^. Recent-onset type two diabetes is defined as those whose diagnosis has been made within a 10-year time frame, within the NICE guidance. Patients with an Asian ethnic background should be considered for bariatric surgery “at a lower BMI than other populations” [5].

A previous study showed that more than 2 million people could be eligible for bariatric surgery in the UK, showing the demand far exceeds the capacity to provide the treatment [6]. The 2014 changes to the guidelines should theoretically lower the threshold for eligibility for bariatric surgery. When this is combined with rising obesity levels [3], this suggests more people should be eligible for bariatric surgery than previously estimated. Despite this, the recent trends in data show that between 2011/2012 and 2014/2015, the number of bariatric surgeries performed in the UK fell from 8794 to 6384, a 27% decrease [7]. Studies have shown bariatric surgery to have a significant impact on obese patients, increasing both life expectancy and quality of life [8]. It has also been shown to be a cost-effective treatment for patients, especially those with type 2 diabetes [9].

This study had a primary objective and two secondary objectives. The primary objective was to determine how many people in England are eligible for bariatric surgery, under the current NICE guidelines. Secondary objectives were to then compare this with the previous guidelines and to compare demographics from the eligible group with demographics of those receiving surgery to examine whether there are health inequalities in current service provision.

## Materials and Methods

### Participants and Data

This was a secondary data analysis of a cross-sectional survey (Health Survey for England). Data was taken from the 2014 survey, downloaded from the UK data archive. The Health Survey for England is designed to be nationally representative by splitting the country into postcode groups before choosing a random sample of households from each group (a stratified probability sample) [10]. In each household, up to ten adults and up to two children are eligible for inclusion in the final dataset. In the 2014 survey, 9024 households were chosen at random from the 564 postcodes. Initial interviews are performed and then followed up by a visit from a trained nurse where possible. Household response rate was 62% to give a total sample size of 8077 adults aged 16 and above and 2003 children aged 0–15. Of these, 5491 adults and 1249 children also had a nurse visit [11].

Participants under 16 were excluded from our study, as were those without an accurate BMI result, or other missing data. Current recommendations state that children and adolescents should only be considered in extreme scenarios and even then surgery should not be performed until they have reached or nearly reached developmental maturity [5]. Not all obesity-related comorbidities are specifically stated in the NICE guidelines so these were determined from what is available in the Health Survey for England and that is considered to be a relevant comorbidity in the guidelines (type 2 diabetes, hypertension) and from the advice of bariatric surgeons (osteoarthritis, cardiovascular disease, stroke, hypercholesterolaemia).

BMI was available in the dataset and had been calculated from measured height and weight, with measurements taken at the interview stage. Type 2 diabetes status was estimated from answers to questions regarding diabetes including age at diagnosis, doctor confirmed diabetes, absence of pregnancy, and gestational diabetes. Undiagnosed cases were identified through an HbA1c blood test obtained at the nurse visit. A participant was considered to have hypertension if they confirmed that a doctor had given them that diagnosis in the past, or if examination of their prescribed medicines identified an antihypertensive medication. Osteoarthritis status was self-reported at the interview stage. Cardiovascular disease, stroke, and hypercholesterolaemia status were self-reported at the interview stage and/or identified by those taking relevant medications (as determined by the Health Survey team).

During the interview stage, participants also self-reported various sociodemographic variables, such as age, sex, ethnicity, income, educational level, and employment status.

### Data Analysis

SPSS 20 was used for computation and analysis of data.

Two sets of initial analyses were performed, one using the new NICE guidelines from 2014 and one using the older 2006 guidelines. The main difference between the two was the recommendation in the 2014 guidelines relating to bariatric surgery at a lower BMI than in the 2006 guidelines for patients with recent-onset type 2 diabetes.

Participants were grouped according to BMI reflecting the ranges of BMI that would be eligible for surgery. For the 2014 guidelines, this was generally non-eligible (< 30 kg/m^2^), eligible with recent-onset type 2 diabetes (30–35 kg/m^2^), eligible with obesity-related comorbidities (35–40 kg/m^2^), and eligible (> 40 kg/m^2^). For the 2006 guidelines, this was non-eligible (< 35 kg/m^2^), eligible with comorbidities (35–40 kg/m^2^), and eligible (> 40 kg/m^2^). In both groups, there were recommendations to consider a lower threshold for the patients with an Asian background. No specific figure was given in the guidelines but the briefing paper published before the guidelines was released suggested a figure of 2.5 kg/m^2^ lower for high-risk (BMI ≥ 30 kg/m^2^) patients with an Asian background [12], so the BMI ranges used for each category were 2.5 kg/m^2^ lower for participants in this group.

Using Boolean operators, participants in the sample were coded as eligible for surgery or non-eligible based on their ethnicity and appropriate BMI category, and the identified comorbidities. This determined the percentage of participants (with a 95% confidence interval) that would be eligible for bariatric surgery in England, calculated using Wilson’s scores with continuity adjustments. Sociodemographic characteristics were determined for the original sample and compared with the eligible population, these were compared where possible with hospital episode statistics data. Frequencies were compared and *p* values calculated using a chi-squared test.

Survey data was weighted to the general population using weighting data supplied with the dataset to account for sampling errors. Where results have been scaled to the general population of England, an estimated figure of 46,550,257 adults (16 or over) is used, based on mid 2014 population estimates and calculated using the 2015 Office for National Statistics (ONS) population data, using the available analysis tool [13].

## Results

### Number Eligible for Bariatric Surgery

There were a total of 6938 adults (over 16) in the sample once those with invalid BMI data were excluded. After weighting, the total sample size was 4996.1, with 388.9 participants eligible for bariatric surgery. This is equivalent to 7.78% (95% CL 7.07–8.58) of the sample. Scaling up for the population of England, an estimated 3,623,505 people would have been eligible for bariatric surgery in 2014.

### Comparison with Old Guidelines

With the same sample but using the eligibility criteria from the previous set of NICE guidelines, 291.7 participants were eligible, equivalent to 5.84% of the population (95% CL 5.21–5.54). Scaling results up to the population of 2014 gives an estimate of 2,717,861 people. This means that the changes made in the new guidelines increased the number that would be eligible for surgery by an estimated 905,644 people or one third.

### Comparison with HES Data for those Receiving Surgery

**Fig. 1:**
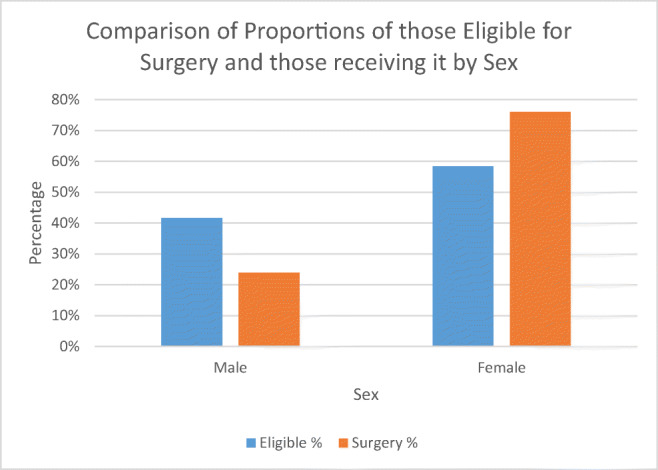
Showing the proportions of those eligible for surgery and those receiving it in 2014 grouped by sex

The results from the sample showed that although a higher proportion of females was eligible for surgery than males (58.4% vs 41.7%), a disproportionately high number received bariatric surgery (76.1% vs 23.9%), as seen in Table 1 and Fig. 1. This was confirmed to be statistically significant (chi-squared = 774.0, *p* = < 0.0001).

**Table 1:** Percentages of those estimated to be eligible and the percentages of those receiving surgery by sex, the weighted figures for who was eligible from the sample, and the numbers that received surgery and the expected number if the proportions of those receiving surgery were equal to the eligible group. Note that due to rounding, totals do not always appear to be exactly 100%

Sex	Eligible %	Surgery %	Eligible	Surgery	Expected
Male	41.7	23.9	161.8	1444	2509
Female	58.4	76.1	227.1	4588	3523
Total	100%	100%	388.9	6032	6032
				Chi-squared	774.0
				*p* value	< 0.0001

**Fig. 2:**
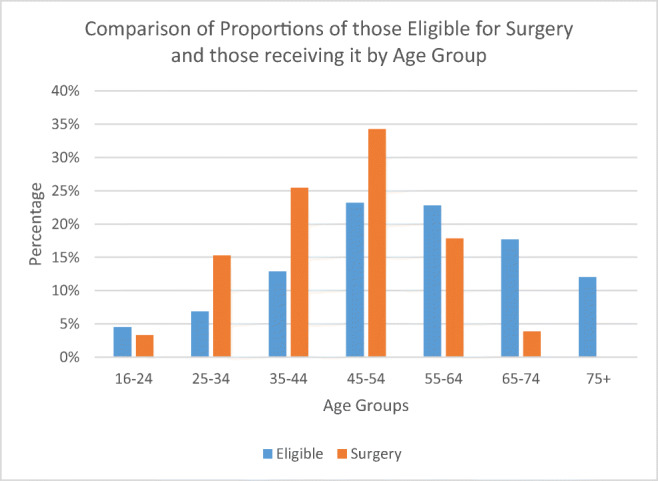
Showing the proportions of those eligible for surgery and those receiving it in 2014 grouped by age group

When split by age group, both those eligible and those receiving surgery peaked at ages 45–54 (23.2% vs 34.2%). There was a higher proportion eligible for surgery over the age of 65 than those that received it (29.7% vs 4.00%) and a lower proportion of those eligible under 35 compared with those that received surgery (11.4% vs 18.6%). Overall, both the eligible population and the surgical population had the majority of cases in the 35–64 age groups, but the overall proportion was lower in the eligible group (58.9% vs 77.5%). There was a significant difference in the distribution of proportions of those eligible for surgery and those who received it (chi-squared = 3115.9, *p* = < 0.0001) as seen in Table 2 and Fig. 2.

**Table 2:** The percentages of those estimated to be eligible and the percentages of those receiving surgery split by age group, the weighted figures for who was eligible from the sample, and the numbers that received surgery and the expected number if the proportions of those receiving surgery were equal to the eligible group

Age	Eligible %	Surgery %	Eligible	Surgery	Expected
16–24	4.5	3.3	17.7	200	274
25–34	6.9	15.3	26.8	920	415
35–44	12.9	25.4	50.1	1532	776
45–54	23.2	34.2	90.3	2064	1399
55–64	22.8	17.8	88.5	1074	1372
65–74	17.7	3.9	68.8	235	1067
75+	12.0	0.1	46.8	4	725
Total	100%	100%	388.9	6029	6029
				Chi-squared	3115.9
				*p* vFweight losalue	< 0.0001

There is a difference in surgery total for sex (6032) and age group (6029). This is due to their being three under 16s who received surgery who could not be removed from the analysis for sex, as raw hospital episode statistics data were not available.

## Discussion

### Main Findings

Results show that under the current NICE guidelines for obesity, an estimated 3,623,505 or 7.78% (95% CL 7.07–8.58) of the population could potentially be eligible for bariatric surgery compared with 2,717,861 or 5.84% of the population under the previous guidance (95% CL 5.21–5.54). These numbers assume that those in the BMI 35–40 category have attempted weight loss already through non-surgical means as required by the NICE guidelines [5]. Although it was not possible to ascertain who in that category had undertaken non-surgical interventions, it is unlikely that many had. However, evaluations of current medical and life style–focused weight loss programmes show that weight loss that is sustained by these programmes is often small, although significant [14]. This means that it would be reasonable to assume that most of those that are eligible would remain eligible even after attempting non-surgical interventions. Certainly, not everyone who is eligible for bariatric surgery should necessarily be operated on. However, as the total number of bariatric surgeries performed in the UK in 2014 was 6032 [7], there is a large difference between potential treatment demand and the availability of bariatric surgery. This penetration rate of 0.002% is particularly low compared with other published penetration rates, for example, 1.24% in the USA (where the eligible population was defined as adults with BMI ≥ 40) [15] and 0.54% in Canada in 2017 (where the eligible population was defined as adults with BMI ≥ 35) [16].

Results also showed that although a higher proportion of those eligible for surgery are female (~ 58% vs 42%), the proportion of females who received surgery is significantly higher (~ 76% vs 24%). There was also a significant difference in the age groups, with the eligible population being skewed towards older age groups and those that receive surgery being skewed towards younger age groups.

There does not appear to be a previous attempt to compare the UK eligible population with hospital episode statistics data. The closest previous study, published in 2014 (but using 2006 data), produced a similar model for estimating who was eligible using the previous NICE guidelines [6]. Their results showed a similar pattern for demographics, although there was a higher proportion eligible in the younger age groups (18–24) and lower in the older age groups (65+) than in this study. The overall estimate for eligible population from that study was 5.1%, which compares closely with this study’s results using the previous guidelines (5.8%), and may partly be explained by considering the increases in obesity prevalence over the intervening eight years.

### Limitations of this Study

The most important strengths and limitations come from the use of the Health Survey for England. This data source is a large, nationally representative sample with trustworthy data including measured heights and weights, and nurse visits to identify, for example—the medication that participants are taking. However, the routine nature of the data collection, rather than a bespoke design specific to our research question means that some variables, we would have ideally had access to, were unavailable. In addition, some conditions were only self-reported, meaning the prevalence of some comorbidities may be underestimated if they are undiagnosed.

Importantly, in terms of variables that were missing, there were several relevant conditions that might indicate surgery would be appropriate that were considered when planning this study, including obstructive sleep apnoea and polycystic ovary syndrome (additional to the comorbidities we did examine: type 2 diabetes, hypertension, osteoarthritis, cardiovascular disease, stroke, and hypercholesterolaemia). However, it was not possible to obtain information on the presence of these conditions from the Health Survey for England. The prevalence of some of the comorbidities chosen may have been underestimated, for example hyperlipidaemia could only be determined from those already taking lipid-lowering medications as there was not enough information to also include those that were untreated but with a raised serum cholesterol. Similarly, it was also difficult to distinguish between type 1 and type 2 diabetes. The Health Survey for England data used a distinction of type 1 being “diagnosed before age 35, treated with insulin” and type 2 being “diagnosed after age 35, not treated with insulin”. This could potentially underestimate the number with type 2 diabetes; however, it was the best way to distinguish between the two conditions with the data available.

The NICE guidelines recommend an assessment for bariatric surgery for those with a BMI of 30–34.9 kg/m^2^ with recent-onset type two diabetes; however, there was not a way to determine which of the cases of type 2 diabetes was recent-onset so again, it is assumed that all those that had type 2 diabetes were eligible. This may overestimate the number eligible through this criterion, although the total number of type 2 diabetes cases is believed to be higher than estimated in this study. This study found the estimated prevalence of diabetes of any type to be 6.1%, whereas diabetes UK figures, based on the QOF data, reported the prevalence of all types of diabetes in England to be around 6.2% in 2014 [17] and Public Health England estimated the number to be around 8.6% [18], with 90% being type 2 diabetes (i.e. 7.7%).

Another possible source of error is a lack of detail in the NICE guidelines. This is presumably to allow for clinical judgement but it presented some difficulties when planning this study. An example of this would be a definitive BMI range for patients from an Asian background, with the guidelines giving the recommendation to consider surgery at a lower BMI range [2, 12]. Another issue was which comorbidities to include, as a definitive list is not provided in the guidelines. There was also a lack of detail at which age surgery would usually be considered. The guidelines do not give an exact lower age, only recommending that surgery not be considered in “children and young people”. In the original study design, eighteen was chosen as a lower limit; however, when reviewing the hospital episode statistics data for 2014/2015, it was decided that this should be lowered to 16 to allow for an accurate comparison, as the lowest age range in the data was 16–24 [7]. There is no stated upper age limit for surgery eligibility, as decisions should be made on a case-by-case basis.

### Implications of this Study

Possible reasons for low demand for bariatric surgery among the eligible population include weight bias, which is high in the UK [19]. Where obesity is attributed to personal irresponsibility, demand, and referrals for bariatric surgery may well be lower. Recent calls from the UK’s Royal College of Physicians [20] to consider obesity a disease might counter any reluctance to encourage bariatric surgery in those patients who would benefit.

Other than the lack of bariatric surgery performed in comparison to demand, the main discussion points from this study would be to determine why there is such a difference in the sociodemographic proportions of who is eligible for surgery and who receives it. The difference in age groups may be due to older age groups being poor candidates for surgery or just an attempt at earlier intervention to prevent chronic comorbidities developing.

With regard to male and female populations, there may be a difference in the proportion seeking medical attention for weight-related issues, as well as concordance with guidance and treatment plans. It is already known that males are less likely to visit health care professionals due to different attitudes towards health care [21]; however, it is not known whether this relates to obesity-related conditions. A previous study in California found that males who presented for bariatric surgery were typically older and with more severe comorbidities [22], showing a greater need for earlier intervention in this group and greater potential benefit from receiving surgery. An area for follow up would be to speak to health care professionals, both in primary care settings to see who seeks help for obesity-related health problems and in secondary care to see who is referred and how surgical candidates are identified. This would help explain why there is such a difference and address any potential barriers to treatment.

Further follow-up work could be undertaken to see if there is a difference between eligibility and treatment in different ethnic groups, different socioeconomic classes, or an urban rural gradient. This would be dependent on the availability of data relating to these groups and bariatric surgery.

## Conclusion

The potential demand for bariatric surgery in England outweighs the availability of services. For a treatment deemed cost-effective [9], increasing uptake of bariatric surgery could benefit the current obesity crisis in England but would require significant investment so that service provision is adequate for demand. There is also a potential health inequality between who requires surgery and who receives surgery, with females receiving a disproportionate number of the available surgeries. Further research is needed to identify if there are any barriers preventing access to treatment for males or whether there is a problem with males seeking treatment for obesity-related health problems and a more proactive approach is needed to identify males in need of treatment.
