# Correction to: An Examination of Who Is Eligible and Who Is Receiving Bariatric Surgery in England: Secondary Analysis of the Health Survey for England Dataset

**DOI:** 10.1007/s11695-023-06495-5

**Published:** 2023-02-13

**Authors:** Daniel Desogus, Vinod Menon, Rishi Singhal, Oyinlola Oyebode

**Affiliations:** 1 Warwick Medical School, Coventry, UK; 2 University Hospitals of Coventry and Warwickshire NHS Trust, Coventry, UK; 3 Heart of England NHS Trust, Birmingham, UK

**Correction to: Obesity Surgery (2019) 29:3246–3251**

**https://doi.org/10.1007/s11695-019-03977-3**

Due to human error, the authors neglected to multiply the rate by 100 in order to get the percentage; therefore, the published percentage for penetration rate was out by a factor of 100.

The correct percentage is included in the paragraph below.

This penetration rate of 0.17% is particularly low compared with other published penetration rates, for example, 1.24% in the USA (where the eligible population was defined as adults with BMI ≥ 40) [15] and 0.54% in Canada in 2017 (where the eligible population was defined as adults with BMI ≥ 35) [16].

